# The expression and clinical significance of serum exosomal-long non-coding RNA DLEU1 in patients with cervical cancer

**DOI:** 10.1080/07853890.2024.2442537

**Published:** 2024-12-17

**Authors:** Yu Chen, Facai Cui, Xiaoyu Wu, Weifeng Zhao, Qingxin Xia

**Affiliations:** a Department of Pathology, The Affiliated Cancer Hospital of Zhengzhou University & Henan Cancer Hospital, Zhengzhou, PR China; b Department of Clinical Laboratory, Henan Provincial People’s Hospital, People’s Hospital of Zhengzhou University, Zhengzhou, PR China; c Department of Oncology, Henan Provincial People’s Hospital, People’s Hospital of Zhengzhou University, Zhengzhou, PR China

**Keywords:** Cervical cancer, long non-coding RNA, lymphocytic leukaemia deletion gene 1, diagnosis, prognosis

## Abstract

**Background:**

Accumulating evidence has demonstrated that the long non-coding RNA (lncRNA) lymphocytic leukaemia deletion gene 1 (DLEU1) is abnormally overexpressed in many cancer types, including cervical cancer (CC). However, the potential clinical significance of DLEU1 in serum exosomes of patients with CC remains unclear.

**Methods:**

The expression of serum exosomal DLEU1 was detected by quantitative real-time polymerase chain reaction (qRT-PCR). A receiver operating characteristic (ROC) curve was plotted to evaluate the clinical diagnostic efficacy of DLEU1. The Kaplan–Meier survival curve and Cox proportional hazards model were used to assess the effect of DLEU1 on postoperative recurrence, metastasis and prognosis among patients with CC.

**Results:**

Our research showed that DLEU1 expression in the serum exosomes of patients with CC was significantly upregulated compared to that in patients with cervical intraepithelial neoplasia (CIN) and healthy controls (HCs) (both *p* < .001). DLEU1 relative expression was significantly correlated with tumour size, cervical invasion depth, pathological grade, International Federation of Gynecology and Obstetrics (FIGO) stage and lymph node metastasis among patients with CC (*p* < .01 all). The combined detection of DLEU1, carbohydrate antigen 125 (CA-125) and squamous cell carcinoma (SCC) exhibited significantly higher diagnostic efficiency (*p* < .01). Furthermore, the overall survival (OS) and disease-free survival (DFS) of CC patients in the high DLEU1 expression group were markedly lower than those in the low DLEU1 expression group (both *p* < .01). Cox univariate and multivariate regression analyses indicated that DLEU1 was an independent risk factor for postoperative recurrence and metastasis in CC patients.

**Conclusions:**

Our findings suggest that serum exosome DLEU1 has certain clinical value for diagnosing, monitoring recurrence and metastasis, and evaluating CC prognosis.

## Background

Cervical cancer (CC), a common malignancy of the female reproductive system, ranks third in incidence and fourth in mortality among all gynaecological cancers [1]. According to the World Health Organization, approximately 600,000 new cases of CC and 340,000 deaths due to CC were reported globally in 2020 [2, 3]. Thus, malignancy is a serious threat to the health of women worldwide. Screening for high-risk human papillomavirus (HPV) and vaccination against HPV are effective measures for preventing CC occurrence. However, its high cost has resulted in low vaccination rates [4]. Serum carbohydrate antigen 125 (CA-125) and squamous cell carcinoma (SCC) antigen tests are commonly used clinical biomarkers for CC screening. These antigens play important roles in the diagnosis, monitoring of treatment effects, and prognosis assessment of CC. However, their low specificity and sensitivity often result in missed diagnosis in some patients [5–7]. Furthermore, delayed treatment may lead to a poor prognosis. Therefore, it is imperative to identify more reliable serum biomarkers with higher sensitivity and specificity.

Exosomes are nano-sized membranous vesicles released into the circulatory system by normal or tumour cells. They regulate the activity of recipient cells and mediate intercellular communication via diverse bioactive molecules they carry, such as small peptides, proteins, nucleic acids and lipids [8]. Long non-coding RNAs (lncRNAs) are a type of non-coding RNAs abundantly present in the cytoplasm and nucleus. They do not encode proteins but regulate gene expression at the epigenetic, transcriptional and post-transcriptional levels [9, 10]. Exosomal lncRNAs play important roles in tumour progression and are potential biomarkers for diagnosing tumours, early warning of recurrence and metastasis, and assessment of disease prognosis owing to their stable expression in body fluids and resistance to RNase degradation [11, 12]. However, reports on the value of exosomal lncRNAs in CC diagnosis are scarce.

In recent years, researchers have found that many lncRNAs are closely related to the diagnosis and prognosis of CC [13]. Of which, lncRNA lymphocytic leukaemia deletion gene 1 (DLEU1), a recently discovered non-coding RNA, its expression in tumour tissue was associated with HPV infection status and served as a biomarker in HPV-infected CC [14]. In contrast, serum DLEU1 has been shown to be a potential prognostic marker for endometrial cancer, another gynaecological tumour [15]. Compared to lncRNAs in tumour tissues, serum exosome-derived lncRNAs have significant advantages in sample acquisition. However, no study has reported DLEU1 expression in serum exosomes of patients with CC or its diagnostic and prognostic value in CC. Therefore, in this study, we focused on investigating DLEU1 expression in serum exosomes obtained from CC patients and its clinical significance.

## Materials and methods

### Clinical data

From 1 June 2018 to 28 February 2019, 134 patients with CC who underwent radical surgery at Henan Provincial People’s Hospital were recruited into the CC group. Concurrently, 50 patients diagnosed with cervical intraepithelial neoplasia (CIN) at the same hospital during the study period were recruited into the CIN group, and 50 healthy individuals undergoing physical examinations were recruited into the healthy control (HC) group. Fasting venous blood (3–5 mL) was collected from all patients in the morning. After centrifugation at 3500 rpm (centrifuge radius: 13.5 cm) for 5 min, the serum was extracted, placed into 1.5-mL centrifuge tubes free of RNase, and stored at −80 °C. Fasting venous blood was collected 6 months after surgery and at the time of recurrence or metastasis, treated in the same manner, and stored at −80 °C after centrifugation.

The inclusion criteria for patients with CC were as follows: (1) the tumour tissue biopsy result was diagnosed as CC by two senior pathologists; (2) patients who did not undergo radiotherapy, chemotherapy or targeted therapy before the surgery; and (3) complete clinical data of patients were available with a signed informed consent form. The exclusion criteria were as follows: (1) previous diagnosis of other malignancies, (2) concurrent primary malignant tumours in other locations and (3) severe organic diseases.

The age of the 134 patients with CC ranged from 27 to 71 years, with an average age of 43.7 ± 12.1 years. Of these, 37, 77 and 20 had poorly, moderately and highly differentiated tumours, respectively. In total, 107 cases of SCC and 27 cases of adenocarcinoma of the cervix were diagnosed. According to the International Federation of Gynecology and Obstetrics (FIGO) staging criteria (2018 edition), 46, 33 and 55 patients were stage I, II and stage III included 55 patients. Furthermore, 79 patients showed no lymph node metastasis, whereas 55 showed lymph node metastasis. Moreover, 71 patients showed a cervical invasion depth of >2/3, whereas 63 patients showed a depth of ≤2/3. Additionally, 25 patients had no HPV infection, 11 had low-risk HPV infection and 98 had high-risk HPV infection. This study was approved by the Ethics Committee of Henan Provincial People’s Hospital (approval number: 2018-126).

### Patient follow-up

Patients with CC included in the study were followed-up via outpatient visits or telephone calls, starting from the first day after surgery until 28 February 2024. Overall survival (OS) was recorded considering death due to CC progression during the follow-up period as the endpoint. Additionally, disease-free survival (DFS) was documented, considering CC recurrence or metastasis during follow-up as the endpoint.

### Isolation and identification of serum exosomes

Exosomes were isolated from serum using a total exosome isolation kit (from serum) (catalogue number: 4478360; Invitrogen, Carlsbad, CA) following the manufacturer’s instructions. Briefly, 1 mL of serum was thawed at 25 °C and centrifuged at 2300 rpm for 30 min to remove the cells and debris. The supernatant was mixed with 200 µL exosome isolation reagent and incubated at 4 °C for 30 min. The solution was centrifuged at 15,000 rpm for 10 min, the supernatant was discarded, and the extracted exosome particles were resuspended in 1× phosphate-buffered saline (PBS) for subsequent experiments. Then, 10 μL of the exosome suspension was placed on a copper grid for 1 min, excess fluid was absorbed with filter paper, stained with 2% phosphotungstic acid for 2 min, and dried at room temperature for 10 min. The characteristics of the serum exosomes were confirmed by transmission electron microscopy (TEM, Thermo Fisher Scientific, Waltham, MA) and nanoparticle tracking analysis (NTA, Zeta View, Particle Metrix, Dusseldorf, Germany).

The levels of exosomal markers TSG101 and CD63 and the endoplasmic reticulum protein calnexin were determined by western blotting. The exosomes were lysed, total protein was extracted using RIPA lysis buffer (Beyotime, Shanghai, China), and the protein concentration was determined using the BCA Protein Assay Kit (Beyotime, Shanghai, China). A total of 50 μg of protein was subjected to sodium dodecyl sulphate-polyacrylamide gel electrophoresis for 1.5 h, and the proteins were transferred onto a polyvinylidene fluoride membrane. The membranes were blocked with 1% bovine serum albumin overnight, incubated with monoclonal rabbit anti-human antibodies against CD63 (1:1000, Proteintech, Wuhan, China), TSG101 (1:5000, Proteintech, Wuhan, China) and calnexin (1:1000, Abcam, Cambridge, UK) for 2 h, washed with TBST, incubated with a goat anti-rabbit secondary antibody at 37 °C for an hour, and then immersed in 1 mL of the developing solution in the dark for approximately 10 min.

### Quantitative real-time polymerase chain reaction (qRT-PCR)

RNA extraction kit, dye-based quantitative PCR kit, and heat-stable reverse transcriptase were purchased from Novogene (Beijing, China). Total RNA from serum exosomes was extracted following the manufacturer’s instructions, and complementary DNA (cDNA) was synthesized by adding total RNA to a reaction system containing reverse transcriptase. The PCR amplification system was prepared, and the amplification conditions were as follows: pre-denaturation at 95 °C for 10 min, followed by 46 cycles at 95 °C for 20 s, annealing at 56 °C for 25 s and extension at 71 °C for 20 s. U6 was used as an endogenous reference gene, and the 2^−ΔΔCt^ method was used to calculate the relative expression of DLEU1. The upstream primer sequence for DLEU1 was 5′-TGCATTTAAAACCGCCCTGC-3′ and the downstream primer sequence was 5′-TTGAAGAAGGAGACCACGCC-3′. The upstream primer sequence for U6 was 5′-GCTTCGGCAGCACATATACTAAAAT-3′ and the downstream primer sequence was 5′-CGCTTCACGAATTTGCGTGTCAT-3′. Both sets of primers were designed and synthesized by Generay Biotech (Shanghai, China).

### Detection of CA-125 and SCC concentrations by the electrochemiluminescence (ECL) method

The assay kits for CA-125 and SCC and their accompanying calibrators were procured from Roche Diagnostics (Basel, Switzerland). The concentrations of CA-125 (ng/mL) and SCC (ng/mL) in the serum of all study participants were determined using the ECL method on a Roche Cobas E602 fully automatic ECL immunoassay analyser.

### Statistical analysis

GraphPad Prism 7.0 (San Diego, CA) and SPSS 20.0 (Chicago, IL) were used for graphing and statistical analyses, respectively. The Kolmogorov–Smirnov test was used to determine whether quantitative data followed a normal distribution. Data with skewed distribution were presented as the median (P25, P75). The non-parametric Kruskal–Wallis test was used to compare multiple groups, whereas the Mann–Whitney *U*-test was used to compare two groups. The diagnostic efficiency of various indicators for CC was analysed by performing the receiver operating characteristic (ROC) curve analysis. A survival analysis was performed using the Kaplan–Meier method and Log-rank test. The Cox proportional hazards regression model was used to analyse factors affecting patient prognosis. *p* < .05 was considered statistically significant.

## Results

### Expression of DLEU1 in cancer tissues and serum exosomes of patients with CC

**Figure 1.**
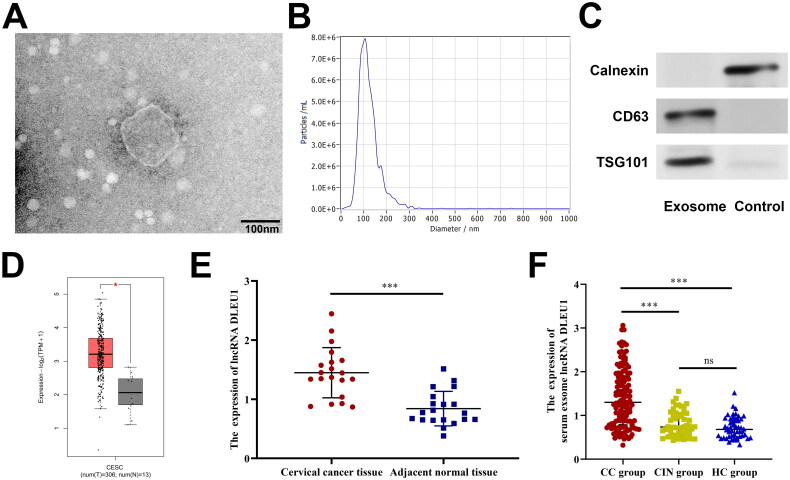
The expression of serum exosomal DLEU1 in patients with CC. (A) The morphological characteristics of exosomes were observed under TEM; (B) the sizes of exosomes were detected using NTA; (C) the expression of TSG101, CD63 and calnexin was detected by Western blotting; D and E: DLEU1 was highly expressed in CC tissues; (F) DLEU1 was highly expressed in serum exosomes of patients with CC. ns: no significant difference. ***p < .001.

We first examined the serum DLEU1 levels in the CC, CIN and HC groups. The results showed no significant difference in the expression of DLEU1 in the serum of the different groups (Figure S1). Therefore, we focused on serum exosomal DLEU1 levels in patients with CC. TEM revealed exosomes with diameters ranging from 50 to 150 nm (Figure 1(A)). Furthermore, NTA measurements revealed that the average size of the serum particles and main peak were approximately 100 nm (Figure 1(B)). Western blotting showed that serum exosomes expressed TSG101 and CD63 but not calnexin, whereas the control group showed the opposite results (Figure 1(C)). These findings confirm the successful isolation of serum exosomes. Analysis using the Gene Expression Profiling Interactive Analysis (GEPIA) 2.0 online database revealed that DLUE1 expression in CC tissues was significantly upregulated compared to that in adjacent non-tumour tissues (*p* < .05), which was further validated by qPCR (*t* = 5.275, *p* < .001) (Figure 1(D,E)). The expression of exosomal DLUE1 was significantly upregulated in the serum of patients with CC compared to that in the remaining two groups (*p* < .001). No significant difference was observed between the HC and CIN groups (Figure 1(F)).

### Relationship between DLEU1 expression in serum exosomes and clinical pathological characteristics of patients with CC

**Figure 2.**
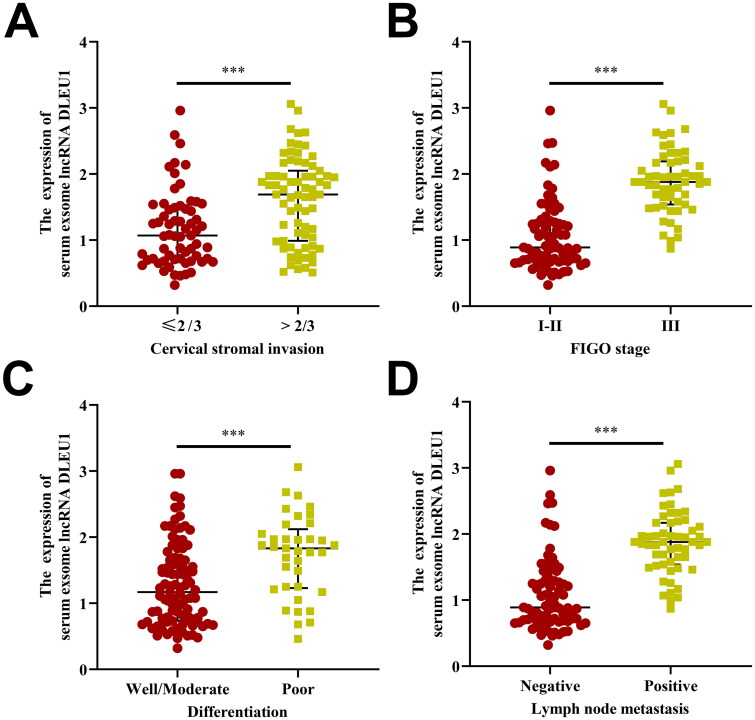
The association between serum exosomal DLEU1 levels and clinicopathological parameters of CC. CC patients with aggressive clinical features (higher cervical stroma invasion (A), positive FIGO stage (B), lower differentiation (C) and positive lymph node metastasis (D)) had higher serum exosomal DLEU1 levels than their respective controls. ***p < .001.

**Figure 3.**
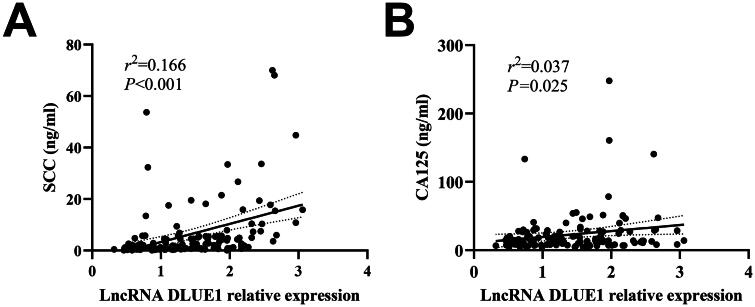
Correlation analysis of the expression of serum exosomal DLEU1 and the concentration of serum SCC or CA-125 in patients with CC. (A) Correlation of the expression of serum exosomal DLEU1 and serum; (B) correlation of the expression of serum exosomal DLEU1 and serum CA-125.

CC patients were grouped according to their clinical characteristics. The Mann–Whitney *U*-test revealed that DLEU1 expression in serum exosomes was associated with tumour size, tumour invasion depth, pathological grade, FIGO stage and lymph node metastasis (*p* < .01 all, Figure 2(A–D)) but was not significantly correlated with patient age, tissue type and high-risk HPV infection (*p* > .05) (Table 1). Furthermore, DLEU1 expression in serum exosomes was significantly positively correlated with serum SCC and CA-125 concentrations (*r*^2^ = 0.166, *p* < .001; *r*^2^ = 0.037, *p* = .025, respectively) (Figure 3(A,B)).

**Table 1.** Relationship between the expression of serum exosomal DLEU1 and clinicopathological features of CC patients *M* (P25, P75).

Clinicopathological features	Case (*n*)	DLEU1 expression	*Z* Value	*p* Value
Age (years)				
≤45	31	1.52 (0.72, 1.96)	0.129	.897
>45	103	1.31 (0.82, 1.88)		
Tumour size (cm)				
≤4	82	1.20 (0.72, 1.71)	2.756	.006
>4	52	1.59 (0.98, 2.17)		
Pathological type				
Squamous carcinoma	107	1.44 (0.81, 1.97)	1.598	.110
Adenocarcinoma	27	0.67 (0.67, 1.64)		
Cervical stromal invasion				
≤2/3	63	1.06 (0.70, 1.52)	4.046	<.001
>2/3	71	1.69 (0.99, 2.12)		
FIGO stage				
I–II	79	0.89 (0.68, 1.29)	7.448	<.001
III	55	1.88 (1.54, 2.19)		
Differentiation				
Well/moderate	97	1.17 (0.74, 1.67)	3.616	<.001
Poor	37	1.83 (1.23, 2.12)		
Lymph node metastasis				
Negative	79	0.88 (0.68, 1.31)	7.219	<.001
Positive	55	1.88 (1.58, 1.19)		
HPV infection type				
Negative/low-risk	36	1.47 (0.90, 1.93)	0.612	.540
High-risk	98	1.28 (0.78, 1.90)		

DLEU1 expression: the relative expression of serum exosomal DLEU1 in CC patients detected by qRT-PCR.

### Analysis of the diagnostic value of serum exosomal DLEU1 combined with CA-125 and SCC for CC

**Figure 4.**
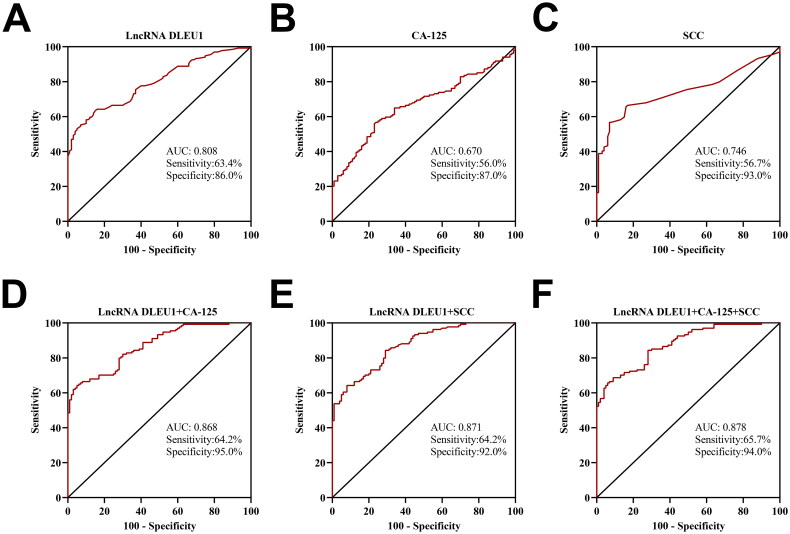
Diagnostic value of serum exosomal DLEU1, serum CA-125, serum SCC alone or in combination for CC. ROC curve analysis of DLEU1 (A), CA-125 (B), SCC (C), DLEU1 + CA-125 (D), DLEU1 + SCC (E) and DLEU1 + CA-125 + SCC (F).

ROC analysis showed that the area under the curve (AUC) for diagnosing CC with DLEU1 in the serum exosomes was 0.808 (95% CI: 0.755–0.862, cutoff value: 1.055), for CA-125 it was 0.670 (95% CI: 0.601–0.738, cutoff value: 13.355), and 0.746 (95% CI: 0.683–0.809, cutoff value: 1.700) (Figure 4(A–C)). When using a single marker for CC diagnosis, the diagnostic efficacy of DLEU1 in serum exosomes was superior to that of CA-125 (*Z* = 2.974, *p* = .003). However, no significant difference was observed in SCC efficacy (*Z* = 1.577, *p* = .115). The AUC for the combined detection of these three markers in diagnosing CC was 0.878 (95% CI, 0.837–0.920; cutoff value, 0.702), which was significantly better than that for detection using serum exosomal DLEU1, serum CA-125 or serum SCC alone (*Z* = 4.833, *p* < .001; *Z* = 6.170, *p* < .001; *Z* = 4.842, *p* < .001) (Figure 4(D–F)).

### Dynamic changes in the relative expression of the serum exosomal DLEU1 and the concentrations of SCC and CA-125 in patients with CC

**Figure 5.**
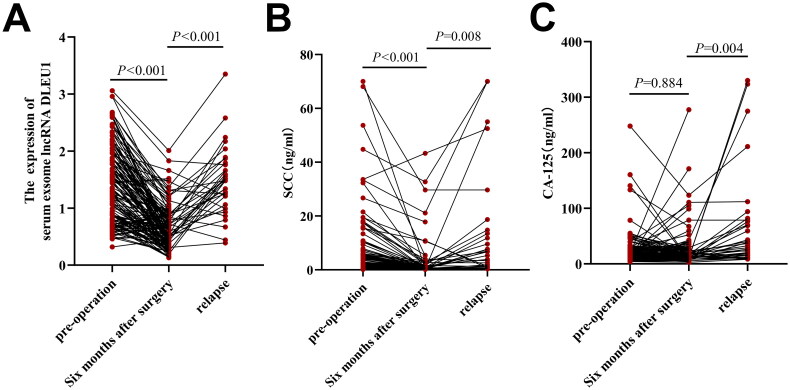
Dynamic changes in serum exosomal DLEU1, serum SCC and serum CA-125 in primary CC patients, postoperative CC patients and recurrent/metastatic CC patients. serum exosomal DLEU1 (A), serum SCC (B) and serum CA-125 (C).

Six months after surgery, the relative expression of DLEU1 and SCC concentrations in patients with CC decreased significantly compared with those before surgery (*p* < .001) (Figure 5(A,B)), whereas CA-125 concentrations showed no significant difference from before and after surgery (*p* > .05) (Figure 5(C)). During the postoperative follow-up of 134 CC patients, 36 cases of recurrence and metastasis occurred. At the time points of recurrence and metastasis, the relative expression of serum exosomal DLEU1, concentrations of SCC, and concentrations of CA-125 were significantly higher than those 6 months after surgery (*p* < .01 all) (Figure 5(A–C)).

### Relationship between serum exosomal DLEU1 expression and prognosis in patients with CC

**Figure 6.**
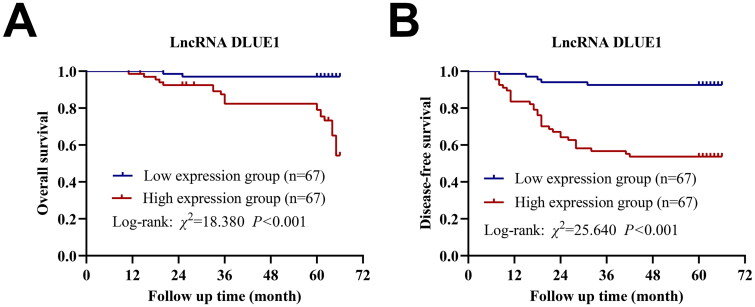
The prognostic value of DLEU1to CC patients. (A) Kaplan–Meier’s survival curves of OS; (B) Kaplan–Meier’s survival curves of DFS.

Based on the median expression, 134 patients with CC were divided into high- and low-DLEU1 expression groups. Kaplan–Meier’s survival analysis indicated that patients in the high-DLEU1-expression group showed shorter OS and DFS than those in the low-DLEU1-expression group (Figure 6(A,B)). Cox univariate regression analysis showed that tumour tissue type, invasion depth, differentiation degree, lymph node metastasis, FIGO stage and relative DLEU1 expression were associated with the prognosis of patients with CC (*p* < .05) (Table 2). Cox multivariate regression analysis revealed that tumour tissue type, differentiation degree and FIGO stage were independent risk factors affecting the prognosis of patients with CC, whereas tumour differentiation degree and relative DLEU1 expression were independent risk factors for postoperative recurrence and metastasis in patients with CC (Table 3).

**Table 2.** Univariate and multivariate Cox proportional hazard analysis of prognosis factor in CC patients.

Variables	Univariate analysis			Multivariate analysis		
	HR	95% CI	*p* Value	HR	95% CI	*p* Value
Age	0.781	0.263–2.320	.656	–	–	–
Tumour size	2.324	0.979–5.517	.056	–	–	–
Pathological type	2.737	1.134–6.606	.025	0.248	0.101–0.610	.002
Cervical stromal invasion	5.872	1.729–19.943	.005	0.491	0.119–2.028	.326
FIGO stage	16.197	3.769–69.608	.000	0.061	0.014–0.268	.000
Differentiation	3.921	1.651–9.313	.002	0.363	0.151–0.871	.023
Lymph node metastasis	16.197	3.769–69.608	.000	0.372	0.018–7.531	.519
HPV infection type	0.585	0.243–1.412	.233	–	–	–
LncRNA DLUE1	10.348	2.409–44.443	.002	0.479	0.081–2.820	.416
CA-125	1.660	0.688–4.005	.260	–	–	–
SCC	1.159	0.492–2.730	.735	–	–	–

HR: hazard ratios; CI: confidence interval.

**Table 3.** Univariate and multivariate Cox proportional hazard analysis of recurrence and metastasis factors in CC patients.

Variables	Univariate analysis			Multivariate analysis		
	HR	95% CI	*p* Value	HR	95% CI	*p* Value
Age	1.561	0.649–3.750	.320	–	–	–
Tumour size	1.447	0.752–2.784	.268	–	–	–
Pathological type	0.589	0.284–1.222	.155	–	–	–
Cervical stromal invasion	0.343	0.161–0.729	.005	1.018	0.392–2.640	.971
FIGO stage	0.255	0.125–0.519	.000	1.057	0.283–3.950	.934
Differentiation	0.195	0.099–0.382	.000	0.269	0.136–0.534	.000
Lymph node metastasis	0.223	0.107–0.463	.000	0.702	0.171–2.882	.624
HPV infection type	0.844	0.415–1.715	.638	–	–	–
LncRNA DLUE1	0.133	0.052–0.343	.000	0.176	0.067–0.459	.000
CA-125	1.416	0.730–2.746	.304	–	–	–
SCC	1.524	0.786–2.957	.213	–	–	–

HR: hazard ratios; CI: confidence interval.

## Discussion

Early symptoms of CC are often inconspicuous, and by the time predominant symptoms are observed, the disease has often progressed to advanced stages with tumours infiltrating the surrounding tissues, accompanied by lymph node metastasis or even distant organ metastasis. This condition complicates surgical treatment and results in a poor prognosis [16]. CC screening is an internationally recognized and effective measure for CC prevention and treatment, which mainly includes liquid-based thin-layer cytology (TCT) and HPV molecular typing. However, the former is characterized by high false-positive and misdiagnosis rates [17, 18], thereby increasing the diagnostic accuracy of pathologists. Compared to TCT, HPV molecular typing exhibits better sensitivity and negative predictive value. However, its lower specificity markedly increases the referral rate for colposcopy [19]. Traditional tumour marker testing is a common method for CC diagnosis. However, its application in clinical settings is limited owing to its low sensitivity and specificity. Exosome testing is a novel liquid biopsy technique [20]. In addition, as lncRNA is a common component of exosomes, it mediates intercellular communication between tumour cells and target cells as well as intracellular signal transduction, thereby playing a crucial role in tumour progression [21]. lncRNAs are protected by the lipid bilayer of exosomes and vesicles. Thus, it is not affected by endogenous RNases and is stable in bodily fluids. Given their temporal- and spatial-specific expression, the importance of exosomal lncRNAs in the diagnosis, treatment and prognosis assessment of tumours is increasingly highlighted.

In previous studies on the progress of exosomal lncRNA and CC, some researchers found that serum exosomal lncRNA-EXOC7 expression was correlated with the FIGO stage of patients and SCC and CYFRA211 concentrations, with a diagnostic AUC of 0.898 for CC [22]. This result implies that exosomal EXOC7 is a good indicator for diagnosing recurrence and metastasis. Ding et al. discovered that high serum exosomal lncRNA DLX6-AS1 expression was associated with lymph node metastasis, degree of differentiation, FIGO stage and patient prognosis; thus, it served as an independent risk factor for patient prognosis [23]. In this study, we identified one lncRNA-DLEU1 present in the serum exosomes of patients with CC. We found that the expression level of CC was closely related to its progression of CC patients.

DLEU1, a newly identified lncRNA, was highly expressed in CC tissues and cells. In the present study, we first validated the differential expression of DLEU1 in cancerous and adjacent non-cancerous tissues of patients with CC, and the findings were consistent with those of previous studies. Further examination of DLEU1 expression in the serum exosomes of patients with CC, CIN and HCs showed that its expression was significantly upregulated in patients with CC and correlated with tumour size, tumour invasion depth, pathological grade, FIGO stage, lymph node metastasis, and SCC and CA-125 concentrations. In a study on the correlation between DLEU1 and CC, some researchers found that DLEU1 was highly expressed in CC cancer tissues and cancer cells. DLEU1 promotes proliferation and migration of CC cells through the miR-381/HOXA13 axis [24]. Dong et al. found that the promoting effect of DLEU1 on malignant progression of CC was related to its mediating effect on multiple miRNAs [14]. In contrast, DLEU1 is considered an immune and necroptosis-related factor, which is closely related to the prognosis of patients with CC [25, 26]. The results of this study further prove that DLEU1 is closely associated with the malignant progression of CC.

In addition to its clinical significance, serum exosomal DLEU1 expression was shown to help dynamically assess CC severity in the present study. The AUC of DLEU1 alone for diagnosing CC was 0.808, with a diagnostic sensitivity and specificity of 61.4% and 86.0%, respectively, which were significantly better than those of traditional tumour markers. The AUC for diagnosing CC with the DLEU1, SCC and CA-125 combination was 0.878, with a diagnostic sensitivity and specificity of 65.7% and 94.0%, respectively, which indicated that combining serum exosomal DLEU1 with traditional tumour markers may remarkably improve tumour diagnostic efficacy. The relative median expression of serum exosomal DLEU1 in 134 postoperative patients with CC was 0.61, which was significantly lower than that at the preoperative stage. In the 36 patients with recurrence and metastasis, the relative median expression of DLEU1 was 1.45, which was significantly higher than that at the postoperative level, suggesting the importance of serum exosomal DLEU1 in assessing patient prognosis for CC. The patients were divided into high- and low-lncRNA-DLEU1-expression groups based on median expression, and Kaplan–Meier’s survival analysis showed that patients in the high-lncRNA-DLEU1-expression group showed shorter OS and DFS rates than those in the low-lncRNA-DLEU1-expression group. Cox univariate and multivariate regression analyses indicated that lncRNA expression was related to recurrence, metastasis and prognosis in CC patients, with DLEU1 being an independent risk factor for postoperative recurrence and metastasis.

However, the present study had certain limitations. First, the sample size was small, and all enrolled patients were from the same hospital. This deficiency leads to deviations between the clinical characteristics of patients and the general situation of the country or region where they are located, thus affecting the reliability of the research results. Therefore, the conclusions of this study need to be validated by enrolling more patients from multiple centres in the future. In terms of molecular mechanisms, it is necessary to explore why DLEU1 promotes the malignant progression of CC *in vivo* and *in vitro*. Finally, novel methods need to be developed in the future to detect serum exosomal DLEU1 expression in CC and other cancers.

## Conclusions

In conclusion, we are the first to demonstrate that serum exosomal DLEU1 overexpression in patients with CC is associated with clinicopathological characteristics and prognosis. Thus, serum exosomal DLEU1 may serve as a potential biomarker for CC diagnosis, early warning of recurrence and metastasis, and prognostic assessment.

## Supplementary Material

Supplemental Material

## Data Availability

The data generated in this study are available upon request from the corresponding author.
