# Supplementary material for: The expression and clinical significance of serum exosomal-long non-coding RNA DLEU1 in patients with cervical cancer
Source: Ann Med. 2024 Dec 17;57(1):2442537. doi: 10.1080/07853890.2024.2442537 (PMC11654034; doi:10.1080/07853890.2024.2442537)

Figure S1. The expression levels of serum DLEU1 in CC patients, CIN patients and HC were measured via qRT-PCR. ns represents no significant difference.


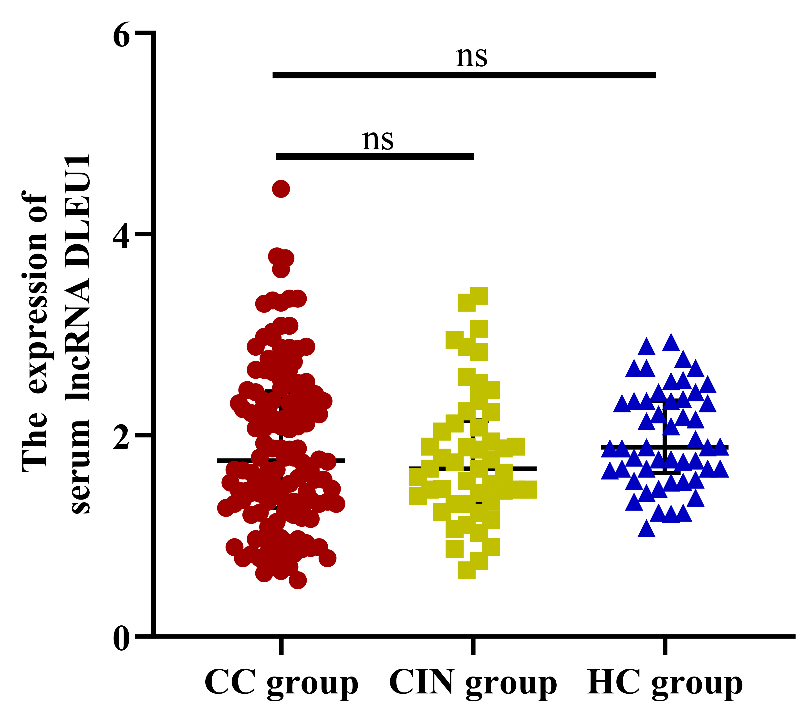

Supplement: Supplemental Material [file IANN_A_2442537_SM7369.docx]
